# Electrochemical control of bone microstructure on electroactive surfaces for modulation of stem cells and bone tissue engineering

**DOI:** 10.1080/14686996.2023.2183710

**Published:** 2023-03-10

**Authors:** Danfeng Cao, Jose G. Martinez, Risa Anada, Emilio Satoshi Hara, Hiroshi Kamioka, Edwin W. H. Jager

**Affiliations:** a Sensor and Actuator Systems, Department of Physics, Chemistry and Biology (IFM), Linköping University, Linköping, Sweden; b Advanced Research Center for Oral and Craniofacial Sciences Dental School, Graduate School of Medicine, Dentistry and Pharmaceutical Sciences, Okayama University, Okayama, Japan; c Department of Orthodontics, Graduate School of Medicine, Dentistry and Pharmaceutical Sciences, Okayama University, Okayama, Japan

**Keywords:** Polypyrrole, plasma membrane, redox switching, bone, chip, organ-on-chip

## Abstract

Controlling stem cell behavior at the material interface is crucial for the development of novel technologies in stem cell biology and regenerative medicine. The composition and presentation of bio-factors on a surface strongly influence the activity of stem cells. Herein, we designed an electroactive surface that mimics the initial process of trabecular bone formation, by immobilizing chondrocyte-derived plasma membrane nanofragments (PMNFs) on its surface for rapid mineralization within 2 days. Moreover, the electroactive surface was based on the conducting polymer polypyrrole (PPy), which enabled dynamic control of the presentation of PMNFs on the surface via electrochemical redox switching, further resulting in the formation of bone minerals with different morphologies. Furthermore, bone minerals with contrasting surface morphologies had differential effects on the differentiation of human bone marrow-derived stem cells (hBMSCs) cultured on the surface. Together, this electroactive surface showed multifunctional characteristics, not only allowing dynamic control of PMNF presentation but also promoting the formation of bone minerals with different morphologies within 2 days. This electroactive substrate could be valuable for more precise control of stem cell growth and differentiation, and further development of more suitable microenvironments containing bone apatite for housing a bone marrow stem cell niche, such as biochips/bone-on-chips.

## Graphical abstract

**Figure:**
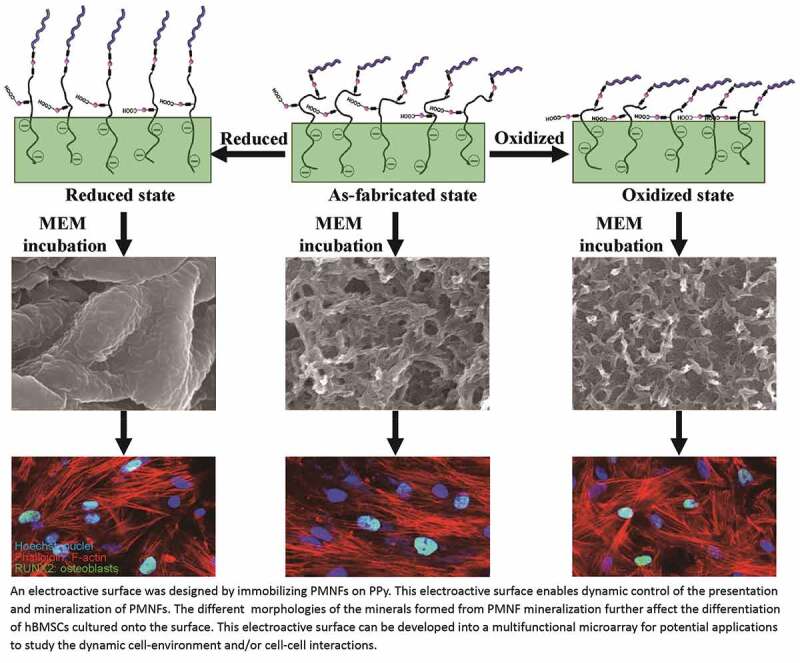

## 1. Introduction

Electro-stimulation (ES) is an effective way to manipulate cellular functions, such as adhesion, growth, and differentiation [1] and has been demonstrated to stimulate bone repair [2,3]. Hydroxyapatite (HAp), the inorganic bone constituent, is known to have a high electrical conductivity [4]. The ES is transduced inter- and intracellularly through the numerous ion channels, pumps, and enzymes contained in the plasma membrane of the cells. Physiological-level ES, which is in the range of mV and µA, is too weak to depolarize the plasma membrane bilipid layer, but is strong enough to activate transmembrane channels and transmembrane receptors [5,6]. Additionally, ES can also be used to modify the presentation of biomolecules, e.g. ligands, that directly affect cellular functions [7–9].

Electrically conducting polymers have attracted much interest on this aspect for the last 20 years because they simultaneously display the physical and chemical properties of organic polymers with interesting properties for ES [1]. The conducting polymers polypyrrole (PPy) and poly(3,4-ethylenedioxythiophene) (PEDOT) have been frequently investigated because of their biocompatibility, electrical conductivity, and redox properties [10,11]. Doped PPy or PEDOT films can be reversibly electrically switched between the different redox states for many cycles, which is accompanied by a reversible injection or ejection of dopants into/from the film. The reversible change of the redox state leads to a reversible change in the surface properties, including surface charges, wettability, and volume [12–14], which can be further exploited to develop electroactive surfaces with ES properties that enable dynamic control of the biological factors, such as proteins [8], eukaryotic cells, and bacteria [15]. Thus, some proteins and drugs can be loaded and released in response to ES [16,17]. These molecules can be doped into the PPy as counterions; however, most of them are too large to be embedded in the polymer matrix as dopants. Even if these macromolecules are doped, it may cause poor conductivity or poor film formation of PPy, and ultimately affect the protein’s function that is needed [18]. Alternatively, surface immobilization of biomolecules on PPy can be used [19]. More recently, covalent binding to conjugated polymer using 1-ethyl-3-(3-dimethylamino) propyl carbodiimide and N-hydroxysulfosuccinimide (EDC/NHS) coupling with carboxylic acid of dopant in the polymer backbone has been done. The EDC/NHS coupling method is a well-established immobilization method for biomolecules that involves mild reaction conditions with little or no influence on the secondary structure of proteins [20,21]. Such electroactive surfaces have immense potential applications, including in the field of biosensors and biochips [22].

*In vitro* biomimetic platforms, including organ-on-chips, are physiological models that enable a detailed analysis of cell-microenvironment and cell-cell interactions, as well as the cellular responses to various external stimuli, such as radiation, drugs, magnetic and electrochemical stimuli, within a tissue or organ [23,24]. Such organ-on-chip models are being developed to obtain a deeper and more precise analysis of cellular responses in many human organs [25], including the liver [26], lung [27], intestine [28], bone [29], blood vessel [30], heart [31], or multiple organs [32] for potential applications in drug development and personalized medicine in cancers and other diseases.

A particular organ is the bone, which is composed of nearly 70% inorganic matter and 30% organic matrix and is the main site enclosing stem cells in adult tissue, the so-called bone marrow niche. However, up to date, there is no *in vitro* biomimetic physiological model, i.e. ‘bone-on-chip’, that replicates the biological function and microarchitecture of bone tissue with cells (e.g. stem cells, osteoblasts, and osteoclasts) and its organic and inorganic matrix components [29].

We have previously shown that plasma membrane nanofragments (PMNFs) act as the nucleation site for bone formation *in vivo* [33]. Moreover, PMNFs isolated from cultured cells could promote mineralization *in vitro* within 2 or 3 days [33,34]. Therefore, PMNFs can be regarded as an excellent bioinspired material to induce rapid bone-like formation on electroactive surface, for instance for use in biomimetic bone-on-a-chip microdevices, compared with conventional osteoconductive materials or other methods using live cells, which in most cases require 2 to 3 weeks to promote mineralization [35].

Here, we used PMNFs as a core material to develop electroactive surfaces mimicking the bone structure as a substrate for primary human bone marrow-derived stem cells (hBMSCs). Moreover, we hypothesized that ES could affect the three-dimensional structure of the PMNFs, and proteins attached to the PPy surface, eventually leading to changes in the PMNF mineralization properties, and changes in the nanostructure of minerals [e.g. amorphous calcium phosphate (ACP), hydroxyapatite (HAp)] formed from PMNF mineralization. In other words, the switchable electroactive polymer surface can dynamically control the presentation of PMNFs, and this switch of PMNFs could result in different mineralizations following the redox switching of the PPy.

**Figure 1.**
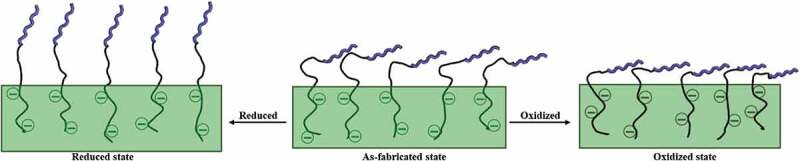
The proposed schematic of the possible change of PMNFs presentation on the PPy surface depending on the redox state of PPy.

Following the rationale of Baumgartner et al. [9], we choose poly-L-glutamic acid (pGlu) as dopant, due to its large number of COOH groups, to ensure that not only the COOH balances the positive charges of PPy originated during electropolymerization but also that some COOH groups on the long pGlu chain would protrude from the surface to couple with the PMNFs. However, PMNFs are large biomolecules composed of numerous proteins and an abundant number of negatively charged phospholipids. Therefore, we decided to introduce L-lysine (Lys) as a linker through two steps of EDC/NHS coupling to bind the PMNFs. We designed this electroactive surface to switch the presentation of PMNFs on the PPy surface to guide the formation of minerals with different morphologies. The proposed schematic is shown in Figure 1. We evaluated how the three redox states of PPy (as-fabricated, oxidized, and reduced) influenced the formation and morphology of minerals formed on the PPy surface and their effects on the differentiation ability of hBMSCs. This approach may lead to new insights into the early-stage bone formation for optimization of methods in bone tissue engineering and would be important to study the interaction of mesenchymal stem cells with different mineral shapes, which in turn, would be critical for further development of more suitable microenvironments containing bone apatite for housing a bone marrow stem cell niche.

## 2. Experimental section

### 2.1. Materials

Pyrrole monomers were acquired from Fluka Sweden, distilled upon arrival, and stored at −20°C until use. Poly-L-glutamic acid sodium salt (pGlu MW 3,000–15,000), L-lysine, N-(3-dimethylaminopropyl)-N’-ethylcarbodiimide hydrochloride (EDC), N-hydroxysuccinimide (NHS), potassium chloride, minimum essential medium (MEM), and toluidine blue O (TBO) were purchased from Sigma-Aldrich and used without further purification. FM®4-64X lipophilic styryl dye was purchased from Thermo Fisher Scientific (Waltham, Massachusetts, USA). Since PMNFs from pre-chondrogenic ATDC5 cells were shown to mineralize faster than those from MC3T3-E1 osteoblastic cells [36], PMNFs were herein isolated only from pre-chondrogenic ATDC5 cells, as reported [33,37]. In brief, the cells were cultured in 225 cm^2^ flasks until confluency, trypsinized, and centrifuged. A total of 1 × 10^7^ cells suspended in 1 mL of ultrapure water were aliquoted in 1.5 tubes and ultrasonicated for 3 min for complete cell fragmentation. The PMNFs were isolated by a series of centrifugation steps at 2.000 g, 10.000 g, and 20.000 ×g, followed by ultracentrifugation at 150.000 g for 60 min. Bicinchoninic acid assay (BCA) was used to indirectly estimate the amount of PMNFs by the concentration of proteins. The isolated PMNFs were freeze-dried and used in the experiments after reconstitution of the lyophilized powder in pure water at a concentration of 100 µg‧mL^−1^.

### 2.2. Preparation of PPy samples

Standard 10 cm silicon wafers were coated with a thermally evaporated 25 Å layer of titanium (for adhesion) and a 1000 Å layer of gold (this substrate is named Au/Si in this paper for simplicity). The wafers were cut into 10 mm × 20 mm pieces, cleaned using TL1 solution (water: H_2_O_2_: NH_3_:H_2_O at the ratio 1:1:5) for 10 min at 80°C and rinsed with distilled water 10 times. Thereafter, a piece of electrical tape limited the area of the wafer to 10 mm × 10 mm and immersed this 1 cm^2^ area was immersed into the electrolyte. Then, PPy was electropolymerized on this 1 cm^2^ Au/Si substrate in 0.1 M Py and 0.002 M pGlu (the concentration of pGlu is based on the glutamic acid unit monomer), using a three-electrode system (3 M NaCl Ag/AgCl reference electrode, stainless steel mesh as counter electrode and Au/Si wafer as a working electrode). The PPy film was generated by applying a constant potential of 0.7 V vs. Ag/AgCl to the working electrode until the consumed charge was 50 mC.

### 2.3. PMNF immobilization onto PPy surfaces

**Figure 2.**
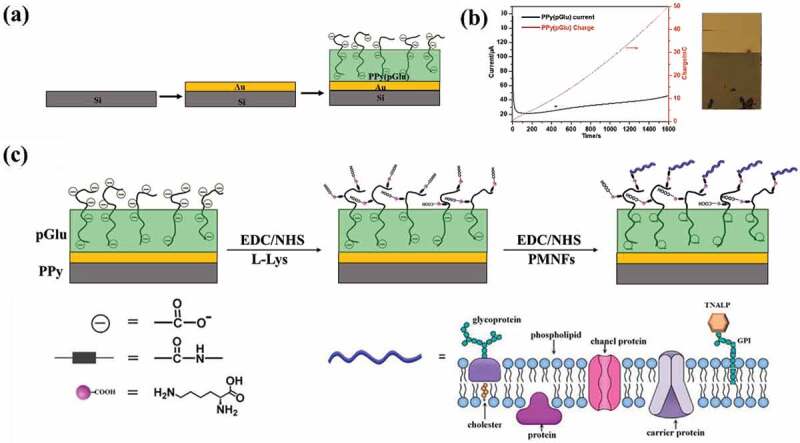
(a) the fabrication process of Au/Si wafer and PPy(pGlu) film on Au/Si wafer; (b) Left, the electrosynthesis current and consumed charge during PPy(pGlu) film synthesis; Right, photograph of the surface: polymerized PPy(pGlu) film on Au/Si wafer; (c) the preparation process of PPy(pGlu)-Lys-PMNF surface.

Subsequently, EDC and NHS coupling chemistry were performed to couple Lys and/or PMNF to the synthesized PPy substrate, as shown in Figure 2(c). A total of 100 µL solution of 0.2 M EDC and 0.05 M NHS with a 1:1 volume ratio was dropped on the PPy surface and maintained for 1 h. The resulting activated surface (PPy(pGlu)-EDC/NHS) was incubated with 100 µL of 4 mg mL^−1^ L-Lys solution for 4 h to allow the formation of the amide bond between the carboxylic group and amine. The EDC/NHS coupling step was repeated to activate the COOH group on the L-Lys to obtain PPy(pGlu)-Lys. The resulting sample was incubated with 100 µL of 100 µg mL^−1^ PMNF dispersion (previously optimized to have uniform and complete coverage of the PPy surface) for 12 h at 4°C to obtain the final product PPy(pGlu)-Lys-PMNF. After each step, the substrate was rinsed with distilled water. The same EDC/NHS coupling method was used to directly couple PMNFs onto PPy(pGlu) surface.

### 2.4. Characterization of PPy films

Electrochemical characterization was used to measure cyclic voltammograms (CV) and electrochemical impedance spectroscopy (EIS) of PPy films using a potentiostat-galvanostat Ivium Stat.XR using the software IviumSoft, Eindhoven, the Netherlands, using the same electrochemical setup as described in 4.2. The CV was done in a 0.2 M KCl solution with a 100 mV s^−1^ scan rate. The EIS of the PPy films was performed at a frequency range of 0.05 Hz −100 kHz in 0.2 M KCl with a bias potential of 0 mV *vs*. the open-circuit potential and a 5 mV amplitude sine wave potential. The redox state of PPy(pGlu)-Lys-PMNF was set by applying a positive (+0.3 V) or negative potential (−0.4 V) to oxidize/reduce the PPy in 0.2 M KCl for 2 h. The impedance spectra were then fitted to an equivalent electrical circuit using aftermath electrochemical studio (1.6.10513 version).

The amount of carboxylic acid groups and PO_4_^−^ negative charge of PMNFs at different immobilization steps of PPy samples were examined via toluidine blue O (TBO) staining assay [38,39]. In brief, the PPy samples were incubated in a 2 mM TBO solution in 0.1 mM KOH for 2 h. Under alkaline conditions, the negatively charged (deprotonated) carboxylic acid groups and PO_4_^−^ groups bind with the positively charged TBO molecules with a ratio of 1:1. Non-specifically bounded TBO molecules on the PPy surface were removed by rinsing with a 0.1 mM KOH solution several times. Finally, the samples were soaked in 5 mL of 50% acetic acid for 30 min to desorb the TBO molecules. Absorbance spectroscopy was performed using a UV – vis spectrophotometer (UV-2450, Shimadzu, Japan) at 631 nm with an established standard calibration curve for TBO concentration vs. absorbance.

Attenuated total reflectance (ATR) was performed with Vertex 70 spectrometer (Bruker, Massachusetts, USA).

Thickness measurements were performed with a Dektak 3ST surface profiler (Veeco Instruments, Germany). The morphologies of all samples were examined with scanning electron microscopy (SEM, Leo 1550 Gemini SEM, Zeiss, Germany).

Water contact angles (WCA) were recorded via a contact angle meter (KVS Instrument, Helsinki, Finland).

The morphology of the samples was also characterized by atomic force microscopy (AFM) using a digital instruments dimension 3100 AFM (USA).

The attachment of PMNFs to PPy was assessed by lipophilic fluorescence staining with 5 μg mL^−1^ FM4–64 dye (N-(3-triethylammoniumpropyl)-4-(6-(4-(diethylamino) phenyl) hexatrienyl) pyridinium dibromide, Thermo Fisher Scientific), for 1 min. The stained samples were observed under a fluorescence microscope (Carl Zeiss™ Axioscope A1 HAL100 Microscope, Germany).

Photoemission experiments were performed with an X-ray photoelectron spectrometer (XPS) using a Scienta® ESCA200 spectrometer in ultra-high vacuum (UHV) with a base pressure of 10^−10^ mbar. The measurement chamber was equipped with a monochromatic Al (Kα) X-ray source providing photons with hυ = 1486.6 eV to measure the results of mineralization.

All the characterizations were performed three times.

### 2.5. In vitro mineralization

The PPy(pGlu)-Lys-PMNF samples were immersed into MEM and submitted to a constant positive potential (+0.4 V, oxidized) or negative potential (−0.5 V, reduced) for 2 h or 2 days continuously. As-fabricated samples were maintained in MEM for the same period. After incubation, the samples were washed with distilled water for 30 min and dried with N_2_.

### 2.6. Cell culture

Primary human bone marrow stem cells (hBMSCs) were purchased from ATCC (Manassas, Virginia, USA). The hBMSCs were cultured in alpha-MEM containing 15% fetal bovine serum (FBS; Sigma-Aldrich, St. Louis, Missouri, USA), 1% penicillin/streptomycin (PS, FUJIFILM Wako Pure Chemical Corporation, Osaka, Japan) at 37°C, 5% CO_2_, and 99% humidity until 80% confluence. Cells at passage P3 were used in the experiments.

For osteogenic differentiation, hBMSCs (5 × 10^3^ cells) were seeded onto the electroactive surfaces (1 cm^2^) and incubated for 24 h to allow cell adhesion. The cells were then maintained in the normal culture medium supplemented with 10 mM β-glycerophosphate (β-GP, Sigma-Aldrich), 100 µM L-ascorbic acid phosphate (Sigma-Aldrich), and dexamethasone sodium phosphate (10^−8^ M), for 14 days [40].

### 2.7. Real-time reverse transcription polymerase chain reaction (real-time RT-PCR) analysis

hBMSCs were cultured in an osteogenic differentiation medium for 14 days onto the as-prepared, oxidized, or reduced PPy(pGlu)-Lys-PMNF surfaces. Total RNA from the cells was collected with trizol (TRI Reagent, Molecular Research Center Inc., Cincinnati, Ohio, USA) and purified using PureLink™ RNA Mini Kit (Thermo Fisher Scientific), according to the manufacturer’s instructions. Concentrations of mRNA were calculated by spectrophotometric measurements using a NanoDrop 2000 spectrophotometer (Thermo Fisher Scientific). Messenger RNA was reverse-transcribed using the iScript cDNA synthesis kit (Bio-Rad, ‎Hercules, California, USA). Real-time RT-PCR was performed using StepOnePlus Real-Time PCR System (Applied Biosystems, Foster, California, USA) using KAPA SYBR green, according to the manufacturer’s instructions. The reference gene s29 was used as the internal control. The primer sequences are as follows: *S29* (sense: TCTCGCTCTTGTCGTCTG; antisense: ACACTGGCGGCACATATTGAGG); *RUNX2* (sense: GACGTTCCCAAGCATTTCAT; antisense: ACTCTGGCTTTGGGAAGAG); *OPN* (sense: ATGTGATTGATAGTCAGGAA; antisense: GTCTACAACCAGCATATCTTCA); and *OCN* (sense: CAGAGTCCAGCAAAGGTG; antisense: AGCCATTGATACAGGTAGC).

### 2.8. Immunocytochemistry

hBMSCs cultured in an osteogenic differentiation medium for 14 days onto the as-fabricated, oxidized, and reduced PPy(pGlu)-Lys-PMNF surfaces were fixed with 4% paraformaldehyde for at least 4 h. The cells were then blocked with 5% goat serum and incubated with primary antibody against RUNX2 (Abcam, Cambridge, UK). The cells were then washed and incubated with goat anti-rabbit IgG conjugated with Alexa Fluor™ 488 for 1 h, at room temperature. The cells were washed and observed under a confocal laser scanning microscope (ZEISS LSM780, Jena, Germany). Nuclei and f-actin were stained with Hoechst® 33342 and phalloidin, respectively. The percentage of cells positive for RUNX2 was measured using pictures from four different areas, by dividing the number of nuclei stained with RUNX2 by the total number of cell nuclei stained with Hoechst. The experiments were repeated with at least three independent samples.

### 2.9. Statistical analysis

Analysis of the differences between groups was performed with a one-way analysis of variance (ANOVA) followed by Bonferroni or Tukey post-hoc tests. All statistical analyses were performed with StatView-5 software (SAS Institute Inc., North Carolina, USA) and GraphPad Prism 9 software (GraphPad Software Inc., San Diego, California, USA). The level of significance was set at *p* < 0.05.

## 3. Results and discussion

### 3.1. PPy synthesis

Figure 2(a) shows the fabrication procedure of PPy(pGlu). Potentiostatic electrosynthesis was used to electrochemically polymerize a PPy(pGlu) thin film on a 10 mm × 20 mm piece of Au/Si wafer using a constant potential of 0.7 V until 50 mC charge was consumed. The electrosynthesis current and consumed charge during PPy(pGlu) synthesis are shown in Figure 2(b). The inset picture in Figure 2(b) is a photograph of the obtained PPy(pGlu) electroactive surface presenting a uniform, greenish-looking film with 200.5 nm thickness on the Au/Si substrate.

### 3.2. Immobilization of PMNFs on PPy and characterization

pGlu was added as a dopant to balance the positive charges of PPy originated during electropolymerization, while a part of the pGlu long chain would expectedly extend outwards the PPy surface to couple with proteins through the COOH groups [41,42]. The reaction procedure is shown in Figure 2(c). The EDC/NHS activated the COOH groups on pGlu to form NHS-ester functional groups. The NHS-ester subsequently reacted with an amino group on Lys to form an amide bond. Next, the resulting PPy(pGlu)-Lys film was again activated with EDC/NHS to immobilize PMNFs, through their amino groups, on the Lys forming PPy(pGlu)-Lys-PMNF. Using the same EDC/NHS coupling, PMNFs were also directly coupled to PPy(pGlu) to compare both methods.

**Figure 3.**
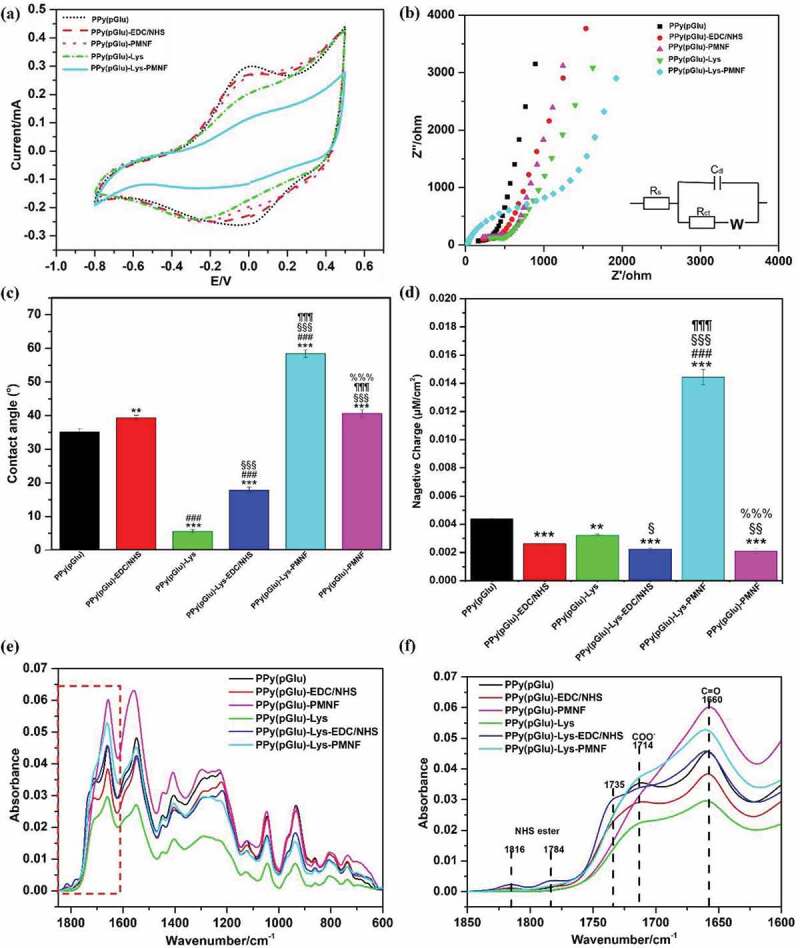
(a) Cyclic voltammograms and (b) results of electrochemical impedance spectroscopy of the different steps of PMNF immobilization onto PPy measured in 0.2 M KCl; (c) Water contact angle results after each immobilization step; (d) Negative charge on the surface during the different steps of immobilization of PMNFs on PPy samples estimated by toluidine blue staining; Attenuated total reflectance results of different steps of immobilization of PMNFs on PPy samples, (e) full scale and (f) enlarged scale. ** *p* ≤ 0.01, *** *p* ≤ 0.001, one-way ANOVA, Tukey test, compared to PPy(pGlu). ### *p* ≤ 0.001, one-way ANOVA, Tukey test, compared to PPy(pGlu)-EDC/NHS. § *p* ≤ 0.05, §§ *p* ≤ 0.01, §§§ *p* ≤ 0.001, one-way ANOVA, Tukey test, compared to PPy(pGlu)-EDC/NHS-Lys. *p* ≤ 0.001, one-way ANOVA, Tukey test, compared to PPy(pGlu)-Lys-EDC/NHS. %%% *p* ≤ 0.001, one-way ANOVA, Tukey test, compared to PPy(pGlu)-Lys-PMNF.

CV and EIS were used to characterize how the electroactivity of the PPy(pGlu) surface changes with the covalent attachments of EDC/NHS, L-Lys, and PMNFs. The CV results in Figure 3(a) characterize PPy samples on Au/Si substrate during various stages of the immobilization procedure in potential scans between −0.8 V and+0.5 V versus Ag/AgCl at 100 mV s^−1^. All samples showed a pair of clearly visible redox peaks indicating good oxidation and reduction of the PPy(Glu) with a current in the range of mA, indicating that the PPy(pGlu), PPy(pGlu)-PMNF, PPy(pGlu)-Lys, and PPy(pGlu)-Lys-PMNF could be reversibly oxidized and reduced so they remained accessible to ions from the electrolyte even though the non-conductive materials of L-Lys and PMNFs were present on the PPy film. The redox peaks of PPy(pGlu)-PMNF still had a similar current as of the unmodified PPy(pGlu) with no peak shift. However, for samples modified through Lys, the oxidation peaks were kept at about 0 V, but the reduction peak potentials of PPy(pGlu), PPy(pGlu)-EDC/NHS, and PPy(pGlu)-Lys-PMNF were shifted, in that order, to the negative potential from 0 V to −0.18 V, to −0.25 V to −0.27 V. The lower reduction peak position while keeping the oxidation peak potential gives a higher separation between the peaks, which indicates a lower conductivity [43], and such lower conductivity can also be observed by the decrease in the current range from PPy(pGlu), PPy(pGlu)-PMNF, PPy(pGlu)-Lys, to PPy(pGlu)-Lys-PMNF following the amount of non-conductive material (PMNFs) immobilized on the surface. Comparing PPy(pGlu), PPy(pGlu)-PMNF, and PPy(pGlu)-Lys-PMNF, the current range of PPy(pGlu)-PMNF only had a minor decrease compared to the unmodified PPy(pGlu), while the PPy(pGlu)-Lys-PMNF decreased notably. This sharp contrast suggested that Lys was critical to immobilize much more PMNFs on the PPy surface. As would be expected, the larger the amount of non-conductive material on the surface, the higher the electrochemical impedance and thus the lower the current.

EIS measurements were taken to further validate the presence of surface groups and characterize how this affects the surface impedance depending on the preparation stage of the PPy(pGlu) film. Figure 3(b) shows the Nyquist plots obtained for the PPy(pGlu), PPy(pGlu)-PMNF, PPy(pGlu)-EDC/NHS, PPy(pGlu)-Lys, and PPy(pGlu)-Lys-PMNF. The semicircle in the Nyquist plots is characteristic of a Randles circuit R_s_(C_dl_[R_ct_Z_W_]) that includes the ohmic resistance of the electrolyte solution (R_s_), the Warburg impedance (Z_W_), the double-layer capacitance (C_dl_), and the electron-transfer resistance R_ct_ [44,45]. As can be seen from Figure 3(b), each EIS of the PPy samples includes a semicircle, which was influenced mostly on the limited electron transfer at higher frequencies and a linear section, which was related to the diffusion (mass transfer) process at low frequencies. As shown in Table S1, after each modification step the R_ct_ of PPy(pGlu), PPy(pGlu)-EDC/NHS, PPy(pGlu)-Lys, and PPy(pGlu)-Lys-PMNF increased. The significant increase in the R_ct_ of the electrode, reflected by the increased semicircle diameter, indicated a slower interfacial electron transport mechanism, which lowered the conductivity of the electrode. The increased R_ct_ of PPy(pGlu)-Lys, PPy(pGlu)-Lys-PMNF, and PPy(pGlu)-PMNF was because the non-conductive Lys and PMNFs coupled to the surface and thus impeded the passage of ions at the interface between the electrolyte and electrode surface (PPy). In addition, the total volume of PMNFs was much larger with Lys, so PPy(pGlu)-Lys-PMNF possesses the highest R_ct_. The R_ct_ of PPy(pGlu)-Lys-PMNF (656 Ω) was higher than PPy(pGlu)-PMNF (255 Ω) and bare PPy(pGlu) (108 Ω), which indicated that the Lys as linker indeed increased the amount of non-conductive material, i.e. a higher density of immobilized PMNFs.

Next, the corresponding surface energy and wettability of the PPy films were determined by static water contact angles (WCA). As shown in Figure 3(c), the PPy samples were all hydrophilic (WCA< 90°). The WCA of PPy(pGlu) (35.1°) and PPy(pGlu)-Lys (5.6°) displayed a lower contact angle than films modified with EDC/NHS, i.e. PPy(pGlu)-EDC/NHS (39.4°) and PPy(pGlu)-Lys-EDC/NHS (17.9°). In addition, the WCA of PPy(pGlu)-Lys-PMNF (58.4°) was the highest compared with all other samples. In line with the literature [46,47], surfaces with high surface energy, e.g. carboxyl content, displayed a strong hydrophilic property. pGlu, containing many hydrophilic carboxyl groups, was embedded into the PPy matrix, but due to its long chain, many carboxyl groups protruded out of the PPy surface. On the other hand, these results can be compared for instance with PPy doped with dodecylbenzene sulfonate, which has no protruding charged groups, and thus, showed a high WCA of ~71.0° [15]. Correspondingly, the WCA of PPy(pGlu)-EDC/NHS (39.4°) and PPy(pGlu)-Lys-EDC/NHS (17.9°) were higher than those of their counterparts without EDC/NHS treatment (PPy(pGlu), 35.1°; PPy(pGlu)-Lys, 5.6°) due to the ester bonding to the carboxylic group. Importantly, Lys grafted on PPy(pGlu) can add one amino and one carboxyl group, meaning that PPy(pGlu)-Lys has more polar groups and consequently a lower WCA compared to PPy(pGlu). Interestingly, the PPy(pGlu)-Lys was almost superhydrophilic, while PPy(pGlu)-Lys-PMNF subsequently showed the highest WCA. This is because PMNFs are mainly composed of phospholipids with long fatty acid chains, generally considered to be low surface energy molecules. Thus, the PPy(pGlu)-Lys-PMNF resulted in the highest WCA, being higher than that of PPy(pGlu)-PMNF, which again indicates a higher amount of PMNFs immobilized by using Lys.

Next, the number of COOH groups on PPy(pGlu), PPy(pGlu)-EDC/NHS, PPy(pGlu)-Lys, and PPy(pGlu)-Lys-EDC/NHS surfaces, as well as COOH and PO_4_- groups on PPy(pGlu)-PMNF and PPy(pGlu)-Lys-PMNF were quantified using the toluidine blue O (TBO) staining technique, which indicates negative charge [38]. Figure 3(d) shows that PPy(pGlu)-Lys-PMNF had the highest amount of negative charge due to the high amount of PO_4_- and COOH groups in the PMNFs. PPy(pGlu) showed the second highest amount of negative charge due to the high amounts of COOH groups in pGlu. Theoretically, in a single-molecule reaction, the amount of COOH groups of PPy(pGlu)-Lys should be similar to that of PPy(pGlu), because Lys modification would consume one carboxyl group but also add a new carboxyl group. However, our results indicate a slight reduction in the number of carboxyl groups in PPy(pGlu)-Lys as compared to PPy(pGlu). This result could be partially explained by the Lys graft efficiency that could be less than the theoretically expected, i.e. possibly not all the COOH groups after being modified with EDC/NHS could be grafted with Lys.

To further determine whether the PMNFs were successfully immobilized on PPy(pGlu) surfaces, attenuated total reflectance (ATR) was performed. Figure 3(e,f) shows the infrared spectra of PPy(pGlu) film and PPy(pGlu) modified with EDC/NHS, Lys, and PMNFs. The carboxylate and amide peaks present in the infrared spectra directly evidence that pGlu was embedded in the PPy film. Some characteristic peaks are present in Figure 3(e): The peak at 3303 cm^−1^ is attributed to N-H in pyrrole and amide groups in Lys and PMNFs. The increased absorption peaks at 1660 cm^−1^ of PPy(pGlu)-PMNF and PPy(pGlu)-Lys-PMNF are amide-I peaks, which are ascribed to C=O vibrations in the amide structure of PMNFs. The absorption at 1714 cm^−1^ corresponds to carbonyl stretching in carboxylic acid groups of pGlu, Lys, and PMNFs. The peak at 1714 cm^−1^ was the highest in the PPy(pGlu)-Lys-PMNF sample, indicating a successful binding of PMNFs. On the other hand, PPy(pGlu)-PMNF did not show a clear carbonyl stretching characteristic peak at 1714 cm^−1^, suggesting that the PPy(pGlu) was grafted only with a few PMNFs due to the lack of Lys as a linker. The evidence of a coupling reaction between carboxylic acid and EDC/NHS can be seen by the formation of amide bonds (NHS ester) [21,22,48]. Figure 3(f) shows the peaks of the activated NHS ester group. The peaks at 1816 cm^−1^, 1784 cm^−1^, and 1735 cm^−1^ are attributed to the carbonyl stretching of NHS ester and a symmetric and asymmetric stretch of NHS carbonyls, respectively. These NHS ester characteristic peaks disappeared when the PPy(pGlu) surface was modified by Lys or PMNFs, which indicated that the activated carboxylic acid groups of PPy(pGlu)-EDC/NHS reacted with the amino groups from Lys or PMNFs to form amide bonds.

Together, these results showed that Lys and PMNFs were successfully grafted onto the PPy(pGlu) surfaces. In addition, since the size of PMNFs is larger than that of the pGlu molecules, the number of PMNFs directly immobilized on PPy(pGlu) could have been limited due to the steric hindrance caused by the limited free space on the surface. Importantly, the number of immobilized PMNFs was greatly improved by introducing Lys as a linker molecule. Lys extended the length of the reactive chain and shifted the PMNF anchoring sites away from the PPy surface.

### 3.3. Mineralization of PMNFs

**Figure 4.**
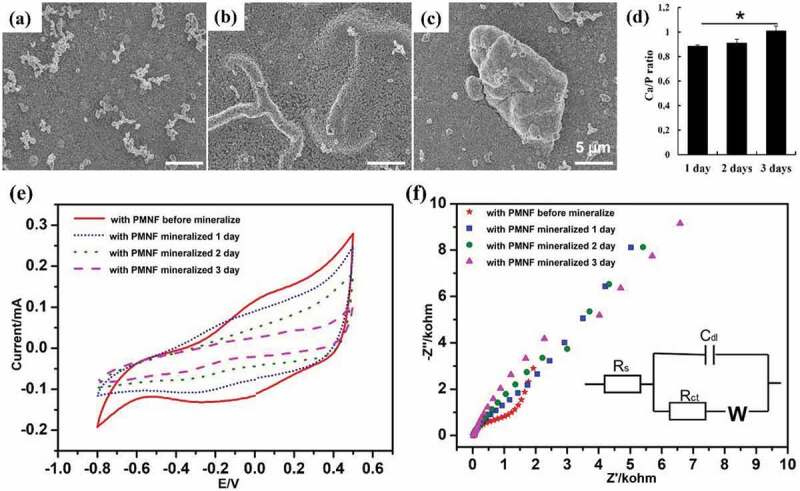
SEM results of PPy(pGlu)-Lys-PMNF in the as-fabricated state after mineralization for (a) 1, (b) 2, or (c) 3 days; (d) the Ca/P ratio of 1–3 days; (e) CV results of PPy(pGlu)-Lys-PMNF after mineralization in MEM for 1, 2, and 3 days, measured in 0.2 M KCl; (f) EIS results of PPy(pGlu)-Lys-PMNF after mineralization in MEM for 1, 2, and 3 days, measured in 0.2 M KCl. * *p* ≤ 0.05, ANOVA, Bonferroni post-hoc test.

The mineralization of PMNFs was first evaluated on as-fabricated PPy samples using SEM, CV, and EIS measurements. Figure 4(a) presents the SEM results of the morphology and the Ca/P ratio of the formed minerals onto the PPy(pGlu)-Lys-PMNF surface after incubation in MEM for 1, 2, and 3 days. The minerals covered the entire surface of PPy after 1 day of incubation. Thereafter, the minerals aggregated to form larger mineral clusters after two or more days of incubation. This confirms that the rapid PMNF mineralization previously demonstrated in petri-dishes [33] or alginate hydrogel [34] was unaffected after the immobilization on the PPy surface. Additionally, the Ca/P ratio also increased with increasing incubation periods (Figure 4(d)). Previously, the initial minerals formed until 3 days of PMNF incubation in MEM were shown to be amorphous calcium phosphate (ACP) [33]. X-ray photoelectron spectroscopy measurements were also performed to determine the composition and state of the elements on the surface. Figure S1(a) shows the typical XPS survey spectra of PPy(pGlu)-Lys-PMNF after preparation. It is observed that the P2p peak is low due to the presence of phospholipids. After mineralization, the Ca2p and the P2p peaks could be detected indicating the formation of calcium-phosphate minerals (Figure S1(b)). As a control group, PPy(pGlu) was also incubated in MEM for 3 days. Surface characterization revealed the cauliflower-like structure peculiar to PPy, and EDX confirmed the absence of P and Ca in the PPy(pGlu) sample (Figure S2).

CVs obtained in 0.2 M KCl aqueous solution from PPy(pGlu)-Lys-PMNF after mineralization in MEM for 1, 2, and 3 days in the as-fabricated state are shown in Figure 4(e). The current passing through the PPy(pGlu)-Lys-PMNF gradually decreased after incubation for 1, 2, and 3 days. Moreover, regarding the redox peaks, although it is hard to clearly point at the exact potential of the peak for longer incubation times (especially for the anodic peak), it is possible to observe how the reduction peak is displaced towards more cathodic potentials for increased incubation time, pointing to a higher separation of the redox peaks and thus, indicating an increased electrochemical impedance due to mineral formation. This was further confirmed by EIS results of PPy(pGlu)-Lys-PMNF after mineralization for 1, 2, and 3 days in the as-fabricated state (Figure 4(f) and Table S2). The EIS results further show that the incubation time influenced the mineralization of PMNFs in MEM on the PPy surface. The electron transfer resistance gradually increased with the incubation time, from 655.53 Ω to 4244.35 Ω (Table S2), indicating a gradual increase in the formation of minerals on the PPy surface. In other words, the longer the incubation time, the larger the amount of minerals formed and the lower the conductivity.

### 3.4. Redox-switching of PPy-PMNF surfaces

The presentation of biomolecules is an important element that affects their biological function [9]. Similarly, we hypothesized that ES could be used to change the presentation of PMNFs and modify their mineralization behavior (Figure 1) [8,9,19]. Hence, the different presentation and behavior of PMNFs on PPy(pGlu)-Lys surface upon redox switching were evaluated by fluorescence staining, AFM, and WCA. The PMNFs-modified surfaces were used in their as-fabricated or reduced or oxidized states by biasing them at −0.4 V, +0.3 V, respectively, for 2 h or 2 days in 0.2 M KCl aqueous solution to change the presentation of PMNFs.

**Figure 5.**
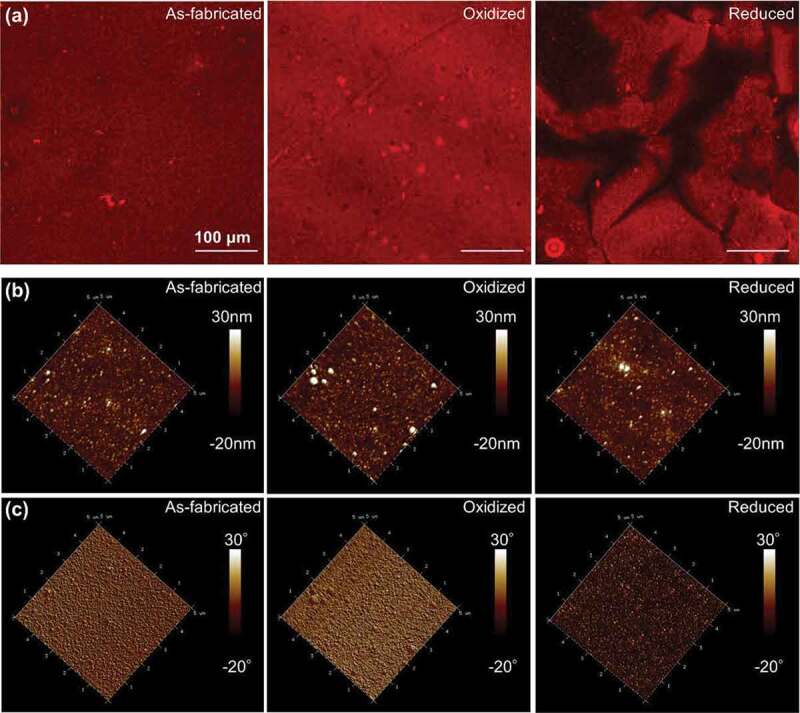
(a) The fluorescence images detecting phospholipids (FM4–64); AFM (b) height and (c) phase images of PPy(pGlu)-Lys-PMNF at as-fabricated, oxidized and reduced states. Redox potential (−0.4 V or 0.3 V) was applied for 2 h.

The fluorescent staining (Figure 5(a)) for phospholipids shows that there is no significant difference between the as-fabricated and oxidized states on the appearance and distribution of PMNFs on the PPy surface. On the other hand, the presentation of PMNFs on the reduced PPy surface showed to be less and the distribution was non-uniform as compared with the surfaces in the as-fabricated and oxidized states. It indicates that the redox switching changed the presentation of PMNFs on PPy surfaces.

In addition, to detect the morphology of PPy(pGlu)-Lys-PMNF at different redox states, tapping mode AFM measurements were performed and their height and phase images are shown in Figures 5(b,c). The height images (Figure 5(b)) do not show much difference between the as-fabricated, oxidized, and reduced states. However, the phase images show a very clear difference between the reduced state and the as-fabricated and oxidized states. The phase of the AFM indicates the surface interaction of the tip and is a measure of the surface energy/properties [49], thus indicating that indeed the PMNF presentation in the reduced state is different from the as-fabricated and oxidized state. In addition, to the general phase difference between the as-fabricated/oxidized and reduced state also red dots appear and exhibit different amounts and sizes on the as-fabricated, oxidized, and reduced surfaces. The red dots on the as-fabricated surface are of medium size and amount. The amount in the oxidized state was the least and the size was the smallest, while the amount and size of red dots in the reduced state exhibit the most and the largest size among these three states. The different presentation of red dots in as-fabricated, oxidized and reduced states further confirmed the different presentation of PMNFs upon switching to the redox state. AFM results clearly show that the redox potential changes the surface properties of the PPy(PGlu)-Lys-PMNF surface. Figure S3 shows the height and phase images of the unmodified PPy(pGlu) in the as-fabricated, oxidized, and reduced states. It also shows no difference among the three conditions, and no red dots were observed onto any of the PPy(pGlu) surfaces. This indicates that the change of the phase is indeed an effect of the different presentation of the PMNFs and not of the redox state of PPy.

**Figure 6.**
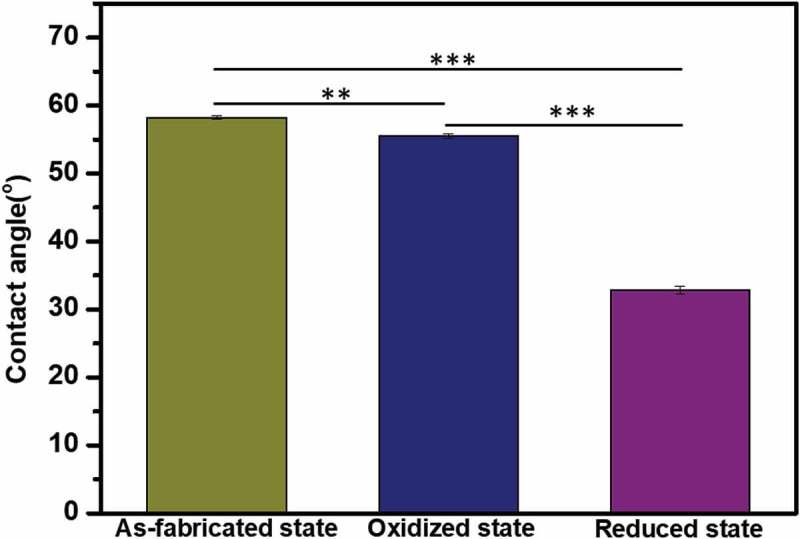
The contact angle of redox state PPy(pGlu)-Lys-PMNF surface. Redox potential (−0.4 V or 0.3 V) was applied for 2 h. ** *p* ≤ 0.01, *** *p* ≤ 0.001, ANOVA, Tukey test.

The surface properties at the three redox states were also evaluated by WCA measurements (Figure 6). The WCA of PPy(pGlu)-Lys-PMNF in as-fabricated, oxidized, and reduced states were 58.2°, 55.5°, and 32.7°, respectively. The WCA of PPy(pGlu)-Lys-PMNF in as-fabricated and oxidized states showed only a slight difference; however, that of PPy(pGlu)-Lys-PMNF in the reduced state was much lower than those of the other two states, presenting a much more hydrophilic surface.

From the above results of WCA at redox switching, we can speculate a change in PMNFs presentation at redox switching. First of all, the wettability change of PPy was controlled by the electrochemical processes that cause ion insertion and de-insertion [50]. Since pGlu is a large, immobile dopant molecule, the doping mechanism can be described as follows:

(1) $$PPy^{n+}\left(pGlu^{n-}\right)+nM^{+}\left(aq\right)+ne^{-}↔PPy^{0}\left(pGlu^{n-}nM^{+}\right)$$

where, M^+^ represents cations in solution, n equals the total number of negative charges on PPy and the number of pGlu dopant into PPy. Hence, when the oxidized potential was applied to PPy(pGlu)-Lys-PMNF, sodium (Na^+^) ions in pGlu were repelled from PPy(pGlu)-Lys-PMNF to maintain charge neutrality, leaving behind embedded long COO^−^ chains in the PPy matrix. Conversely, cations from the electrolyte enter into PPy to maintain charge neutrality when the reduction potential was applied to PPy(pGlu)-Lys-PMNF. Due to the high pGlu molecular weight, part of the long pGlu chain would extend out of the PPy surface and thus play important roles in the surface morphology, surface charge and electron transfer, and interactions with biological systems, as evidenced in section 3.2. In the reduced state, the surface of PPy attracts the K^+^ (from KCl electrolyte) due to coulombic forces to neutralize the PPy film, thus, rearranging Lys-pGlu chains with the negatively charged carboxyl groups away from the PPy surface giving the extended part of the pGlu more freedom. In addition, the more hydrophilic surface of the reduced state can exhibit antifouling properties, resisting to the adsorption of proteins and cells. Hence, this antifouling property may contribute to pushing out the Lys and PMNFs away from the PPy surface altering the PMNF presentation [51,52]. On the other hand, in the as-fabricated and oxidized states, the PPy film could attract more COO^−^ and PO_4_^−^ groups from the pGlu close to PPy film, because the PPy^+^ needs a negative charge to maintain charge neutrality. Thereby, the applied electrical potential would induce the PMNFs to ‘collapse’ onto the PPy surface due to the attractive coulombic forces and the lower hydrophilicity would benefit the adhesion of proteins and thus of PMNFs [53]. The applied electrical potential did not induce formation of lipidic membranes, and the PMNFs remained individual molecules.

### 3.5. The effect of redox switching on PMNF mineralization

**Figure 7.**
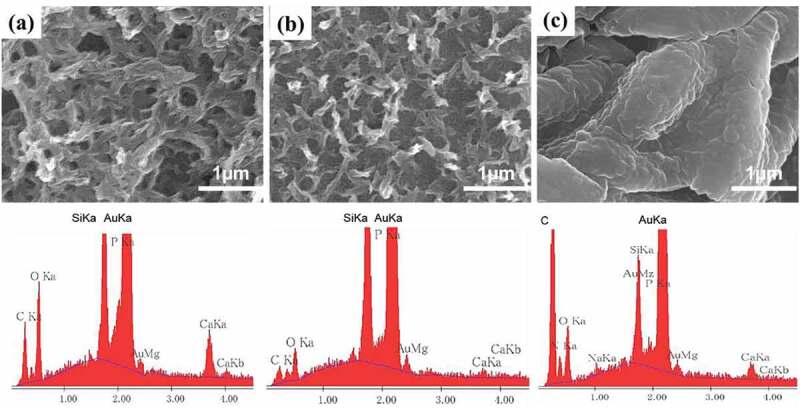
Characterization by SEM and EDX of the minerals formed from PMNF mineralization onto PPy(pGlu)-Lys-PMNF (a) as-fabricated, or in (b) oxidized or (c) reduced states, after incubation in MEM for 2 days.

Next, the PMNF-modified surfaces were incubated in MEM to induce mineralization. To investigate the effect of the redox state (as-fabricated, oxidized, and reduced states) on PMNF mineralization, the samples were incubated in MEM by applying +0.3 V (oxidized), −0.4 V (reduced) or without any applied potential (as-fabricated) for 2 days. The morphology of the minerals was analyzed by SEM (Figure 7). All PMNF samples after being submitted to the different redox states were covered by a mineralized structure. The morphology of the mineral layer on the as-fabricated and oxidized states was similar, showing a three-dimensional mesh-like structure, resembling in part the trabecular bone. However, the minerals in the reduced state formed large bulk structures, completely distinct from those formed in the other two states. The EDX results also indicated that the Ca and P element peaks appeared after MEM incubation. It is worth noting that a short-term application of redox potentials for 2 h, followed by incubation in MEM for 2 or 3 days, did not induce any modification in the mineral morphology (Figure S4). Future works should also investigate the effect of reversible redox switching or other intermediate redox states on the morphology of bone microstructure after PMNF mineralization.

**Figure 8.**
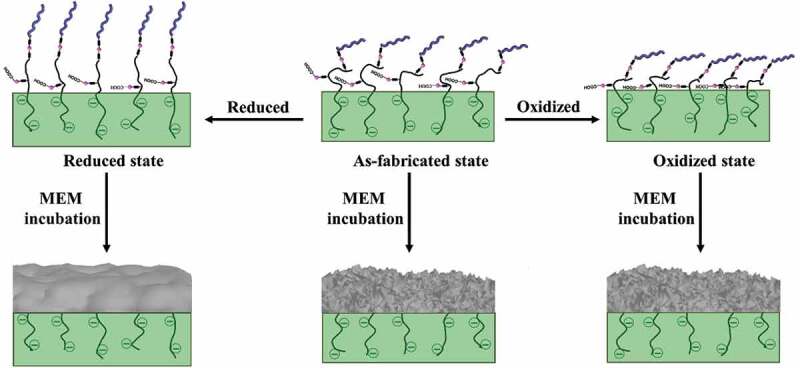
The proposed schematic of PMNFs presentation change on PPy surface and mineralization at as-fabricated, oxidized and reduced surfaces.

The following events could be associated with the ES-induced changes in the PMNF mineralization. Based on the principle of switching the presentation of PMNFs controlled by dynamic electroactivity of PPy surface [8,9,19], the oxidized state could induce a collapse of PMNFs, which would now be closer to the PPy surface, and have altered interaction with biomolecules in the medium. On the other hand, at reduced state, PMNFs could be presented away from the PPy surface, and since the long pGlu chain is flexible, PMNFs could be extended unrestrictedly and be able to interact much more with biomolecules in the medium, leading to the formation of large mineral clusters. The schematic is proposed in Figure 8.

### 3.6. Modulation of stem cell differentiation ability by redox switching of PMNF mineralization

**Figure 9.**
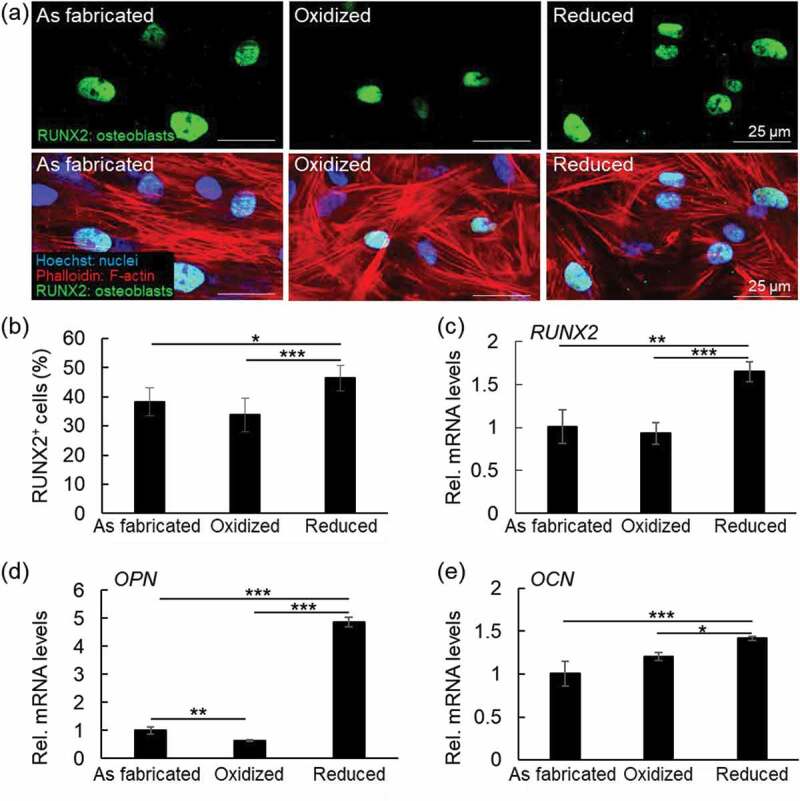
Osteogenic differentiation of hBmscs cultured onto PPy(pGlu)-Lys-PMNF as-fabricated, oxidized or reduced states, after incubation in MEM 2 days. (a) Fluorescence images of hBmscs stained with RUNX2 (upper panel) and merged images (lower panel); (b) Quantitative analysis of the percentage of RUNX2+ cells based on immunofluorescence images. (c-e) Expression levels of (c) *RUNX2*; (d) *OPN* and (e) *OCN* in hBmscs after 14 days of culture in osteogenic differentiation medium. Note the enhanced osteogenic differentiation of hBmscs when cultured onto PPy(pGlu)-Lys-PMNF reduced state. * *p* ≤ 0.05, ** *p* ≤ 0.01, *** *p* ≤ 0.001, ANOVA, Bonferroni post-hoc test.

We then analyzed the effect of the different PPy surfaces (as-fabricated, oxidized, and reduced), including the microstructure of the formed mineral clusters, on the osteogenic differentiation of hBMSCs. As shown in Figure 9, hBMSCs cultured onto PPy(pGlu)-Lys-PMNF in the reduced state showed enhanced osteogenic differentiation compared to the oxidized or as-fabricated counterparts, as demonstrated by the higher percentage of cells immunostained for RUNX2 (Figure 9(a)) and higher mRNA expression levels of *RUNX2*, *OPN,* and *OCN* (Figure 9(b–d)), which are, respectively, early, intermediate, and late osteogenic differentiation markers. Although the levels of the early and late osteogenic markers, RUNX2 and *OCN*, respectively, were not dramatically enhanced, there was indeed a remarkable increase in the intermediate osteogenic marker gene, *OPN*, which is reasonably appropriate considering the induction period of 2 weeks.

A previous study investigated the effect of different surface morphologies of hydroxyapatite minerals on the proliferation and differentiation of hMSCs [54]. Cell expansion was shown to be more intense when hMSCs were cultured onto minerals with a super-large granule-like morphology (granule size >40 μm) compared to plate-like or mesh-like morphologies [54]. On the other hand, osteogenic differentiation of hMSCs, assessed by alkaline phosphatase activity, was significantly lower when the cells were maintained on granule-like minerals compared to those cultured on plate-like or mesh-like minerals for 8 days. In contrast, the results of the present study indicate that mineral clusters of approximately 1 μm significantly enhanced the osteogenic differentiation of hBMSCs, compared to mesh-like minerals formed in the as-fabricated or oxidized states. This differential effect of granule-like minerals onto the osteogenic differentiation of hBMSCs could be attributed to the size difference between the super-large granule-like mineral clusters (>40 μm) and those formed by the PMNF mineralization (≈1 μm), which is in fact close to the morphology of the minerals formed in the early stages of bone marrow formation [55].

These results indicate that hBMSCs can sense and respond differently to the different microstructures of apatite mineral clusters. Interestingly, although the structure of the minerals formed in as-fabricated and oxidized surfaces was comparable to the trabecular bone and could thus be considered more ‘natural’, the mineral clusters formed in the reduced surface in fact promoted much more the osteogenic differentiation of hBMSCs than those in the other two surfaces. Therefore, this bioinspired electroactive surface could further be explored to boost the current methods to induce osteogenic differentiation of hBMSCs for rapid bone tissue engineering and bone repair. PPy has been proposed for use as an electroactive material in medical devices and has passed basic biocompatibility testing following ISO standards, including cytotoxicity, irritation, acute systemic toxicity, and hemolysis [50,56]. These electroactive surfaces could be used in biochip/organ-on-chip platforms to allow a deeper understanding of the interactions of stem cells with the trabecular bone-mimicking microenvironments, which will be critically important for the future development of more suitable microenvironments containing bone apatite for housing a bone marrow stem cell niche.

## 4. Conclusions

We fabricated an electroactive surface comprising PPy doped with pGlu onto which PMNFs were successfully immobilized using EDC/NHS coupling and Lys as a linker molecule. We also demonstrated that by altering the electrochemical potential, i.e. the redox state, we can switch the presentation of the attached PMNFs. The different presentation of the PMNFs at the different redox states influenced the bone-like mineral deposition abilities during incubation in MEM, as seen in the different surface morphology of the formed minerals. Moreover, the different microstructural morphology of the formed minerals was further shown to modulate differentially the osteogenic differentiation of hBMSCs.

In conclusion, this electroactive surface affords an effective approach to provide different functions at different stages, i.e. we can dynamically switch the conformation of covalently attached PMNFs at programmed time points and thereby affect the surface morphology of minerals after MEM incubation and modulate stem cell differentiation in a predetermined way opening up for new tools in (bone) tissue engineering. This electroactive surface can be further developed into a more complex multifunctional biochip/organ-on-chip platform by micropatterning the surface in multifunctional segments forming a microarray. Each electrode (or ‘pixel’) could be modified with various biological factors or using other electrochemically responsive materials modified with biomolecules to create spatial and temporal controllable microenvironments for potential applications to study the dynamic cell-environment or cell-cell interactions in *in vitro* bone (or other organ) models. This smart biochip/organ-on-chip may also lead to enhance the understanding of the early stages of bone formation for optimization of the methods in bone tissue engineering and optimization of the microenvironments containing bone apatite for housing a bone marrow stem cell niche.

## Supplementary Material

Supplemental Material
