# Supplementary material for: Electrochemical control of bone microstructure on electroactive surfaces for modulation of stem cells and bone tissue engineering
Source: Sci Technol Adv Mater. 2023 Mar 10;24(1):2183710. doi: 10.1080/14686996.2023.2183710 (PMC10013253; doi:10.1080/14686996.2023.2183710)
Supplement: Supplemental Material [file TSTA_A_2183710_SM1685.docx]

Supporting information

**Electrochemical control of bone microstructure on electroactive surfaces for modulation of stem cells and bone tissue engineering**

Danfeng Cao^a^, Jose G. Martinez^a^, Risa Anada^b,c^, Emilio Satoshi Hara^b^*, Hiroshi Kamioka^c^, Edwin W. H. Jager^a^*

^a^ Sensor and Actuator Systems, Department of Physics, Chemistry and Biology (IFM), Linköping University, 58183 Linköping, Sweden;

^b^ Advanced Research Center for Oral and Craniofacial Sciences Dental School, Graduate School of Medicine, Dentistry and Pharmaceutical Sciences, Okayama University, 700-8525 Okayama, Japan;

^c^ Department of Orthodontics, Graduate School of Medicine, Dentistry and Pharmaceutical Sciences, Okayama University, 700-8558 Okayama, Japan.

Table S1. EIS analysis of different step samples

| Samples | R_s_ (Ω) | R_ct_ (Ω) | C_dl_ (µF) | W (kΩ/S^0.5^) |
| --- | --- | --- | --- | --- |
| PPy(pGlu) | 1.25 | 1.08 10^2^ | 1.96 | 2.43 |
| PPy(pGlu)-EDC/NHS | 1.90 | 1.81 10^2^ | 9.10 10^-1^ | 3.29 |
| PPy(pGlu)-Lys | 1.85 | 2.31 10^2^ | 1.44 | 2.35 |
| PPy(pGlu)-Lys-PMNF | 2.11 | 6. 56 10^2^ | 1.76 10^1^ | 3.27 |
| PPy(pGlu)- PMNF | 1.67 | 2.55 10^2^ | 1.89 | 2.38 |

Table S2. EIS result of PPy(pGlu)-Lys-PMNF after mineralization in MEM.

| Samples | R_s_ (Ω) | R_ct_ (Ω) | C_dl_ (µF) | W (kΩ/S^0.5^) |
| --- | --- | --- | --- | --- |
| PPy(pGlu) -Lys-PMNF prepared | 2.11 | 6. 56 10^2^ | 1.76 10^1^ | 3.27 |
| PPy(pGlu) -Lys-PMNF incubation 1 day | 2.55 10^1^ | 7.33 10^2^ | 1.65 10^1^ | 7.55 |
| PPy(pGlu) -Lys-PMNF incubation 2 day | 4.22 10^2^ | 1 52 10^3^ | 3.10 10^1^ | 9.42 |
| PPy(pGlu) -Lys-PMNF incubation 3 day | 2.92 10^1^ | 4.24 10^3^ | 4.94 10^1^ | 1.11 10^1^ |


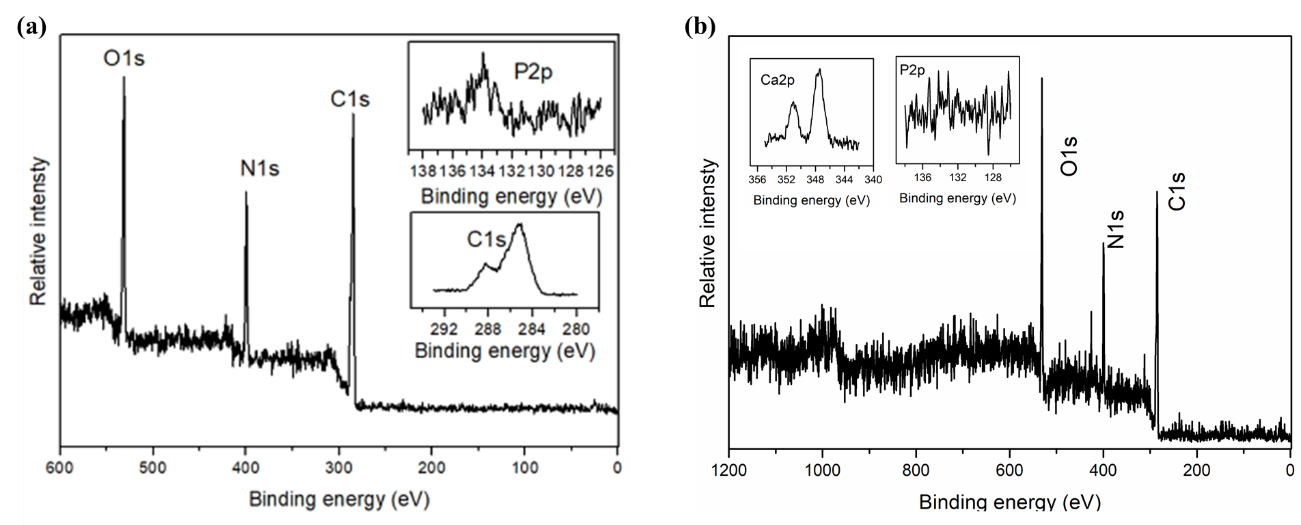


Figure S1. XPS result of as fabricated PPy(pGlu)-Lys-PMNF sample (a) before and (b) after incubation in MEM for 3 days. Note the detection of the calcium peak in (b) only.


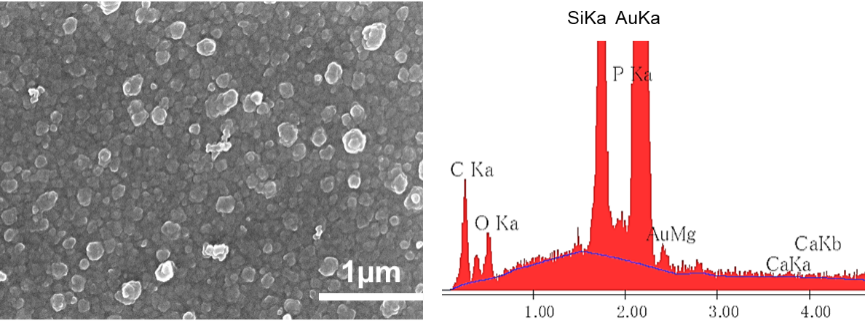


Figure S2. SEM result and EDX result of PPy(pGlu) at as fabricated state after MEM incubation for 3 days. Note the absence of calcium peak in the EDX analysis.


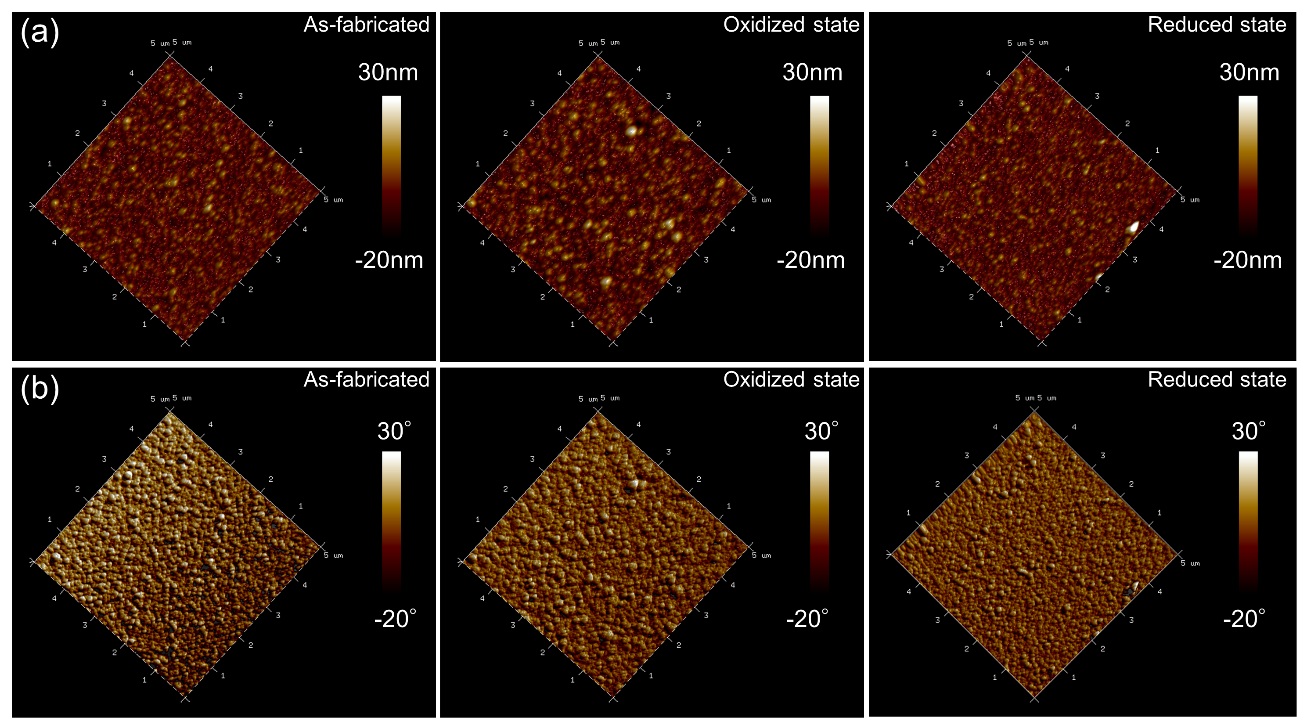


Figure S3. AFM (a) height and (b) phase images of PPy(pGlu) samples at as-fabricated, oxidized or reduced states.


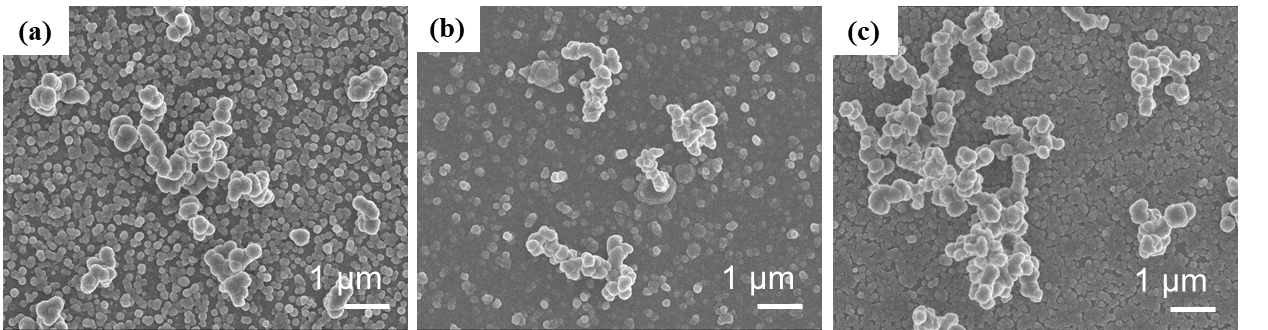


Figure S4. SEM photograph of PPy(pGlu)-Lys-PMNF surfaces after application of redox potential for 2 h and subsequent incubation in MEM for 3 days: (a) as-fabricated, (b) oxidized, and (c) reduced states.
